# Survey data to unveil the power of political crowdsourcing on social media

**DOI:** 10.1016/j.dib.2024.110758

**Published:** 2024-07-16

**Authors:** Rehan Tariq, Izzal Asnira Zolkepli, Pradeep Isawasan, Chekfoung Tan, Muna Mohammad Alhammad

**Affiliations:** a School of Communication, Universiti Sains Malaysia, Pulau Pinang, Malaysia; b College of Computing, Informatics and Mathematics, Universiti Teknologi MARA, Perak Branch, Malaysia; c Centre for Systems Engineering University College London, London, UK; d Department of Management Information Systems, King Saud University, Riyadh, Saudi Arabia

**Keywords:** Political communication, Partisanship, Social capital, Political trust, Social networking sites

## Abstract

This paper describes a dataset collected from a survey carried out in the United Kingdom, Malaysia, and Pakistan, to understand the variables that impact political trust. The data was collected from September to November 2021 via an online survey on Google Forms, and 472 valid responses were obtained. Drawing on relevant literature, the survey instrument was designed to cover the respondents' opinions concerning partisanship, social media utilization, online social capital, voluntary online and offline political participation, and political trust. The dataset offers useful insights for institutional practitioners and policymakers working in the domains of democracy and political communication, facilitating policy formulation to bolster political trust through collaborative crowdsourcing.

Specifications Table

**Table:** 

Subject	Communication
Specific subject area	Social media and political communication
Data format	Raw Data (.csv), Analysed, Descriptive
Type of data	Table
Data collection	Data was acquired via a self-administered structured questionnaire. To enhance comprehension of the scale among respondents and overcome language barriers, the scale was translated from English into Urdu, and Bahasa Melayu, the native languages of Pakistan and Malaysia respectively. The questionnaire was distributed using Google Forms employing snowball sampling. It comprised two distinct sections: Section A contains demographic data, while Section B comprises the instruments employed. Quantitative data was collected from participants’ responses using a 5-point Likert scale. Over a three-month period (September-November 2021), 472 valid responses were received. The questionnaire and an Excel (.csv) data file can be accessed in the repository.
Data source location	The United Kingdom Malaysia Pakistan
Data accessibility	Repository name: Mendeley Data Data identification number: 10.17632/rsd9bcjb2f.1 Direct URL to data: https://data.mendeley.com/datasets/rsd9bcjb2f/1

## 1 Value of the Data

• The data yields insights into the effects of social media activities as tools for enhancing voluntary participation among voters in the UK, Malaysia, and Pakistan, expanding on the Motivation-Incentive-Activation-Behavior (MIAB) model as political crowdsourcing model.
• The data presented here can assist policymakers in devising efficient strategies to enhance political crowdsourcing on social media, fostering political trust among citizens.
• Other scholars can use this dataset to compare developed and developing democratic countries, expanding upon statistical analysis using techniques such as multigroup analysis.

## 2 Background

This research aims to analyse data collected from voters who also engaged with social media, to evaluate their level of political trust. The uniqueness of this data lies in the theoretical foundation provided by MIAB model within the domain of political crowdsourcing. The data will also be used to bridge the gap in the existing literature concerning crowdsourced politics via social media communication. In addition, it holds significance as a mechanism for exploring partisanship, social media utilization, and online social capital under the mechanism of collaborative crowdsourcing to foster voluntary online and offline political participation, ultimately establishing political trust.

## 3 Data Description

Data was collected from the United Kingdom, Pakistan, and Malaysia. The UK is a north-western European parliamentary-based constitutional monarchy. The Malaysian federation is a Southeast Asian parliamentary-based constitutional monarchy [1], and Pakistan is a South Asian republic based on parliamentary democracy [2]. The democratic systems these three countries follow are rooted in the Westminster parliamentary model, which originated in Britain [3]. We selected the UK, Malaysia, and Pakistan for two reasons. Firstly, Malaysia and Pakistan were British colonies and are now commonwealth members, and secondly, they all apply a Westminster-derived structure of government formation [1,4]. Meanwhile variables of partisanship, social media utilization, online social capital, voluntary online and offline political participation, were incorporated to evaluate their influence on political trust, which is an essential political ingredient that ensure the inclusivity of the public policies proposed by political institutions [5]. Theoretically, this article strengthens the findings of the recent research [6,7], drawing on the unique political and technological affordances of social media by exploring its contribution to the quality of democracy.

Data collection was accomplished by implementing a survey methodology, to obtain both demographic information and responses to close-ended questions. Within the specified time frame of September to November 2021, 472 participants actively engaged with the survey, utilizing the Google Form platform. The demographic characteristics of the respondents are illustrated in Table 1. The respondents were asked to use a 5-point Likert scale to express their agreement with statements on social media utilization, online social capital, voluntary online and offline political participation, ranging from 1 to 5, where 1 denotes “Strongly Disagree” and 5 indicates “Strongly Agree’. In contrast, items linked with partisanship were assessed using a scale ranging from 1 to 5, where 1 represents “Weak” and 5 indicates “Extremely Strong”. The raw data contains feedback from the respondents.

**Table 1:** Descriptive statistics for the respondents.

	Frequency			Percentage		
Country of origin	UK	Malaysia	Pakistan	UK	Malaysia	Pakistan
	160	159	153	33.9	33.7	32.4
**Gender**						
Male	91	88	113	56.9	55.3	73.9
Female	69	71	40	43.1	44.7	26.1
**Age**						
18-28	31	10	135	19.4	6.3	88.2
29-39	45	58	16	28.1	36.5	10.4
40-50	34	62	1	21.2	39	0.7
51-60	35	14	0	21.9	8.8	0
Above 60	15	14	0	9.4	8.8	0
Prefer not to answer	0	1	1	0	0.6	0.7
**Education**						
Less than high school	2	1	0	1.2	0.6	0
High School	12	9	3	7.5	5.7	1.9
Vocational	9	5	0	5.6	3.1	0
Bachelor's degree	60	71	71	37.5	44.7	46.4
Master's degree	47	49	74	29.4	30.8	48.4
PhD degree	27	17	5	16.9	10.7	3.3
Prefer not to answer	3	7	0	1.9	4.4	0
**Marital Status**						
Single	51	51	129	31.9	32.1	84.3
Never married	11	2	0	6.9	1.2	0
Married/ civil partnership	81	96	23	50.6	60.4	15
Divorced	8	3	0	5	1.9	0
Widowed	1	1	0	0.6	0.6	0
Separated	1	0	0	0.6	0	0
Prefer not to answer	7	6	1	4.4	3.8	0.7
**Religion**						
Islam	27	51	150	16.9	32.1	98
Christianity	50	2	0	31.3	1.2	0
Buddhism	4	96	0	2.5	60.4	0
Hinduism	4	3	0	2.5	1.9	0
Judaism	2	1	0	1.2	0.6	0
Atheism	31	0	1	19.4	0	0.7
Prefer not to answer	42	6	2	26.2	3.8	1.3
**Employment**						
Full-time	116	101	25	72.5	63.5	16.3
Part-time	11	5	4	6.8	3.1	2.6
Self-employed	13	20	11	8.1	12.6	7.2
Homemaker	1	1	1	0.6	0.6	0.7
Student	11	9	91	6.9	5.7	59.5
Retired	3	15	0	1.9	9.4	0
Disabled- Unable to work	0	0	0	0	0	0
Unemployed but looking for work	2	2	14	1.3	1.3	9.2
Unemployed but not looking for work	1	0	3	0.6	0	1.9
Prefer not to answer	2	6	4	1.3	3.8	2.6

## 4 Experimental Design, Materials and Methods

Data was collected quantitatively applying the inclusion criteria, aged 18 years and above having voted in at least one general election, as the voting age in the target countries is 18 years. Corresponding with the principles of informed consent, potential participants were advised their participation was voluntary and that they were permitted to withdraw from the study at any time. Over a three-month period, 511 responses were received, of which 472 were validated. The online survey questionnaire comprised 46 measurement items for the six latent variables under investigation (see Table 2). The original version of the scale was in English. To develop a better understanding of the scale among respondents, it was translated from English into Urdu and Bahasa Melayu, the native languages of Pakistan and Malaysia respectively, to overcome the language barrier. A multilingual questionnaire ensures the understanding of respondents from different cultures with different education levels. This research instrument was translated and retranslated following the back-to-back translation procedure [8]. The items were adapted from past literature [[9], [10], [11], [12], [13], [14], [15], [16], [17], [18], [19]]. During the validation process, the items were reviewed by an expert panel and statistically tested by the pilot study.

**Table 2:** Variable, conceptualization, and source.

Variables	Conceptualization	Code	Item	Source
Partisanship	Partisanship is a sense of closeness, attachment, and identification towards a particular political party.	PS1	To what extant do you practically support any political party?	[[9], [10], [11]]
		PS2	To what extent do you feel that you are closer to a specific political party?	
		PS3	How strong is your level of association to a specific political party?	
		PS4	To what extent being a partisan is important to you?	
		PS5	To what extent party identity is important to you?	
		PS6	When talking about your political party how often do you use “we” instead of “they”?	
		PS7	To what extent do you find your party affiliation stable during the election campaign?	
		PS8	To what extent do you find your party affiliation stable during the time of voting?	
		PS9	To what extent do you find your party affiliation stable after the election results are announced?	
Social Media Utilization	Social media utilization refers to the intentional and frequent use of social media to seek, share, and understand political issues.	SMUT1	I use social media to get information about current political events.	[12]
		SMUT2	I use social media to get information about current public affairs.	
		SMUT3	I use social media to stay informed about the local community.	
		SMUT4	I use social media to get information about government policies.	
		SMUT5	I use social media to get information about current events from mainstream news social media site.	
		SMUT6	I use social media to get information about current events through friends and family.	
		SMUT7	I use social media to stay informed and get updates during elections.	
		SMUT8	I use social media to discuss political topics.	
		SMUT9	I use social media to stay informed about the people who are politically active on social media.	
Online Social Capital	Online social capital refers to developing a connection with people across particular political interest groups.	OSC1	Interacting with people online makes me interested in things that happen outside of my interest area.	[13]
		OSC2	Interacting with people online makes me want to try new things.	
		OSC3	Interacting with people online makes me feel like part of a larger social community.	
		OSC4	Interacting with people online makes me feel connected to the world.	
		OSC5	I am willing to spend time to support online community activities.	
		OSC6	Interacting with people online let me meet new people to talk to.	
Voluntary Online Political Participation	Voluntary online political participation refers to voluntary participation in diverse online political activities.	VONP1	I voluntarily participated in a political activity online.	[[14], [15], [16]]
		VONP2	I created and invited others to participate in an event related to a political event or social cause.	
		VONP3	I signed an e-mail or web petition that support political events or social cause.	
		VONP4	I forwarded a political e-mail or link to another person.	
		VONP5	I signed up online as a volunteer for political campaign.	
		VONP6	I made a donation to a political party or an organization through online sources.	
		VONP7	I list my political ideology on my social media.	
		VONP8	I made online groups of people to send and receive political updates.	
Voluntary Offline Political Participation	Voluntary offline political participation refers to voluntary participation in diverse offline political activities.	VOFP1	I volunteered for a campaign or other political cause.	[15,17]
		VOFP2	I organized or participated in a political event.	
		VOFP3	I participated in demonstrations or protests.	
		VOFP4	I displayed a political button, sign or sticker.	
		VOFP5	I voted in an election.	
		VOFP6	I tried to influence how others would vote.	
		VOFP7	I got involved in public interest groups, political action groups, political clubs, or party committees.	
Political Trust	Political trust refers to the confidence and trust on political institutions.	PLT1	The government of my country can be trusted.	[18,19]
		PLT2	The police in my country can be trusted.	
		PLT3	The courts in my country can be trusted.	
		PLT4	The judiciary system in my country can be trusted.	
		PLT5	The election commission of my country can be trusted.	
		PLT6	The local government of my country can be trusted.	
		PLT7	Politicians running the government in my country can be trusted.	

We employed a snowball sampling technique to access participants for data collection purposes. This technique is recognized for its utility in accessing hard to reach populations, and for swiftly gathering data [8]. It began by connecting personal contacts and groups on social media platforms that were most closely associated with the target population. Some individuals from the contact list were asked to share the questionnaire among their contacts, and to request that they do the same. To ensure the respondents met the inclusion criteria a filter question was added at the start stating, “Do you use social media, living in the UK, Malaysia or Pakistan, belong to the age group 18 years and above, and have experience voting in General elections?”. Table 2 illustrates the measurement items, while Table 3 provides an assessment of validity and reliability.

**Table 3:** Convergent validity and reliability assessment.

Variables	Items	Loading	Mean	SD	(α)	CR	AVE	ρA
Partisanship	PS1	0.733	3.09	1.384	0.960	0.966	0.758	0.966
	PS2	0.913						
	PS3	0.907						
	PS4	0.890						
	PS5	0.897						
	PS6	0.884						
	PS7	0.892						
	PS8	0.870						
	PS9	0.837						
Social Media Utilization	SMUT1	0.812	2.38	1.294	0.944	0.952	0.690	0.944
	SMUT2	0.850						
	SMUT3	0.781						
	SMUT4	0.844						
	SMUT5	0.773						
	SMUT6	0.892						
	SMUT7	0.887						
	SMUT8	0.794						
	SMUT9	0.837						
Online Social Capital	OSC1	0.855	2.07	0.959	0.921	0.938	0.717	0.923
	OSC2	0.870						
	OSC3	0.853						
	OSC4	0.864						
	OSC5	0.811						
	OSC6	0.827						
Voluntary Online Political Participation	VONP1	0.809	3.48	1.267	0.939	0.950	0.702	0.940
	VONP2	0.790						
	VONP3	0.881						
	VONP4	0.838						
	VONP5	0.855						
	VONP6	0.835						
	VONP7	0.840						
	VONP8	0.854						
Voluntary Offline Political Participation	VOFP1	0.866	3.40	1.406	0.949	0.959	0.769	0.951
	VOFP2	0.844						
	VOFP3	0.928						
	VOFP4	0.876						
	VOFP5	0.922						
	VOFP6	0.797						
	VOFP7	0.898						
Political Trust	PLT1	0.852	2.80	1.064	0.923	0.93	0.655	1.024
	PLT2	0.854						
	PLT3	0.740						
	PLT4	0.739						
	PLT5	0.849						
	PLT6	0.873						
	PLT7	0.744						

## Limitations

None.

## Ethics Statement

The study was exempted from ethical review and approval due to the absence of any ethical issues, such as the inclusion of vulnerable groups, the acquisition of sensitive information, exposure to distressing situations, intrusive activities, or the collection of biological materials related to medical research.

## CRediT Author Statement

**Rehan Tariq:** Writing –original draft, Conceptualization, Formal analysis, Methodology. **Izzal Asnira Zolkepli:** Supervision, Writing –review & editing, Funding acquisition. **Pradeep Isawasan:** Methodology, Project administration, Data curation. **Chekfoung Tan:** Investigation, Writing –review & editing, Resources. **Muna Mohammad Alhammad:** Validation, Writing –review & editing, Conceptualization.

## Data Availability

Political Crowdsourcing on Social Media - Survey Data (Original data) (Mendeley Data).
